# The Tomato *SlVIPP1* Gene Is Required for Plant Survival Through the Proper Development of Chloroplast Thylakoid Membrane

**DOI:** 10.3389/fpls.2020.01305

**Published:** 2020-08-26

**Authors:** Rosa Micol-Ponce, Manuel García-Alcázar, Carmen Capel, Fernando Juan Yuste-Lisbona, Benito Pineda, Alejandro Atarés, Begoña García-Sogo, Juan Capel, Vicente Moreno, Rafael Lozano

**Affiliations:** ^1^Centro de Investigación en Biotecnología Agroalimentaria (BITAL), Universidad de Almería, Almería, Spain; ^2^Instituto de Biología Molecular y Celular de Plantas (UPV-CSIC), Universidad Politécnica de Valencia, Valencia, Spain

**Keywords:** *SlVIPP1*, tomato, chloroplast, thylakoid membrane, *PspA*, albinism, lethality

## Abstract

Since membranes play essential roles in all living beings, all cells have developed mechanisms for efficient and fast repair of membrane damage. In *Escherichia coli*, the Phage shock stress A (PspA) protein is involved in the maintenance of the integrity of its inner membrane in response to the damage produced by exposure to stress conditions. A role in thylakoid membrane maintenance and reorganization has been proposed for Vesicle Inducing Protein in Plastid 1 (VIPP1), the putative PspA ortholog in *Arabidopsis thaliana*. While some membranes of plant cells have been extensively studied, the biosynthesis and maintenance of chloroplast thylakoid membrane remains poorly known. Here, we report the cloning and functional characterization of the tomato (*Solanum lycopersicum* L.) ortholog of *Escherichia coli PspA* and *Arabidopsis thaliana VIPP1*, which we dubbed *SlVIPP1*. Our genetic and molecular characterization of *slvipp1*, an insertional mutant, allowed us to conclude that the tomato *SlVIPP1* gene is needed for development, as Arabidopsis *VIPP1*, but not *Escherichia coli PspA*. Homozygous *slvipp1* tomato plants are albino and exhibit early lethality and highly aberrant chloroplast development with almost complete absence of thylakoids. The phenotype of tomato RNAi lines and that of additional *slvipp1* alleles generated by CRISPR/Cas9 gene editing technology confirmed that the morphological and histological aberrations shown by *slvipp1* homozygotes are caused by *VIPP1* lack of function. We also found that tomato *SlVIPP1* overexpression does not cause any visible effect on plant morphology and viability. Our work with *slvipp1* plants evidences that *SlVIPP1* is an essential gene required for tomato survival, since its function is crucial for the proper formation and/or maintenance of thylakoid membranes.

## Highlights

Evidence of the prokaryotic origin of plant chloroplasts include the existence of plant nuclear genes whose products play similar functions in photosynthetic bacteria and plant chloroplasts. Tomato *SlVIPP1*, like its Arabidopsis *VIPP1* ortholog, is one of such genes, whose studies in both model species reveal that the formation and maintenance of thylakoid membranes is an essential process for plant growth and survival. Interestingly, *VIPP1* homologous genes are highly conserved among plants and between plants and oxygenic photosynthetic bacteria.

## Introduction

Life on Earth depends upon atmospheric oxygen, which is produced by cyanobacteria and plants through oxygenic photosynthesis. Oxygen production is a crucial and highly efficient process that uses solar energy to convert carbon dioxide and water into sugars and other organic compounds, hence providing resources for plant and animal life. Oxygenic photosynthesis occurs in the thylakoids, membrane-bound compartments consisting of an ordered internal membrane network surrounding a thylakoidal lumen. The photosynthetic machinery, including the photosystems I and II (PSI and PSII) and the light-harvesting complexes I and II (LHCI and LHCII), is embedded in the thylakoid membranes. Chloroplasts of land plants differ from those of green algae in the building of an intertwined network of stroma lamellae and grana lamellae, thylakoids being the structural units of this lamellar system. In addition, chloroplasts are surrounded by a double-membrane envelope (reviewed in Mechela et al., 2019).

The bacterial phage shock protein (Psp) response was discovered as a mechanism of response to phage infection in *Escherichia coli* (Brissette et al., 1990). In the Psp response of *Escherichia coli* six proteins are involved: PspA to PspE, encoded by the *pspABCDE* operon, which is regulated by the transcriptional activator PspF, encoded by a gene placed upstream the operon (Jovanovic et al., 1996). During the phage infective process, the PspA protein is continuously synthetized at high levels, leading to much higher concentrations than the other Psp proteins. PspA plays a dual role, inhibiting transcription of the *pspABCDE* operon by sequestering PspF, and binding to the PspB-PspC complex and to the bacterial cytoplasmic membrane, likely to provide stress relief. PspA is also induced by other abiotic stresses as heat or high osmotic shock. Although the Psp response was discovered as a mechanism of bacterial envelope integrity maintenance, Psp-like proteins are not exclusive to bacteria, since they also exist in archaea and plants (Joly et al., 2010; Flores-Kim and Darwin, 2016).

A PspA-like protein was identified in *Pisum sativum* as a 37-kDa precursor, whose processing results into a mature protein of 30 kDa. This protein was termed Inner Membrane-associated 30-kDa protein (IM30) because it exhibited a dual localization, associated to the chloroplast envelope and thylakoid membrane (Li et al., 1994). A T-DNA insertional mutant allowed the identification of Arabidopsis *VESICLE-INDUCING PROTEIN IN PLASTIDS 1* (*VIPP1*), the first plant homolog of *Escherichia coli PspA* functionally characterized. The Arabidopsis VIPP1 protein has been localized at the chloroplast envelope and the thylakoid membrane, and its depletion causes a pale-green phenotype in cotyledons, which are deficient in photosynthesis due to its poor thylakoid membrane development (Kroll et al., 2001). It was later found that Arabidopsis VIPP1 is required for thylakoid membrane formation but not for the assembly of protein complexes in thylakoid membranes (Aseeva et al., 2007). In addition, VIPP1 function in heat stress is known to be essential for membrane protection (Zhang et al., 2016). It has also been described that Arabidopsis VIPP1 forms extremely large homo-oligomeric rings and interacts with the inner envelope membrane of chloroplasts (Vothknecht et al., 2012; Zhang and Sakamoto, 2013; Zhang and Sakamoto, 2015). Although VIPP1 does not contain canonical GTP-binding domains, it is likely to be a novel type of GTPase, acting in chloroplast membrane fusion and/or remodeling, due to its capability for GTP binding and hydrolysis (Ohnishi et al., 2018). However, a recent study in *Synechocystis* suggest that the Vipp1 capability for GTP hydrolysis, is not essential for its membrane remodeling function (Junglas et al., 2020). Two VIPP1 proteins are encoded by the genome of *Triticum urartu*, and the heterologous expression of one of these proteins, TuVipp1, rescues the phenotypic effects of Arabidopsis *vipp1* (Gao et al., 2017).

In the unicellular cyanobacteria *Synechocystis*, Vipp1 has been detected at the thylakoids, the plasma membrane and the cytoplasm, and its loss causes disruption of thylakoid membrane development, as in Arabidopsis (Srivastava et al., 2005; Srivastava et al., 2006; Fuhrmann et al., 2009). *Synechocystis* Vipp1 has a paralog, PspA, which lacks a C-tail of 30 amino acids, and results more similar in structure to *Escherichia coli* PspA than to Arabidopsis VIPP1 (Westphal et al., 2001). It was initially thought that cyanobacteria *VIPP1* was an essential gene, required for thylakoid membrane biogenesis (Westphal et al., 2001; Gao and Xu, 2009). However, the isolation of a viable *vipp1* null mutant suggests that *Synechococcus* Vipp1 is not essential, being required for biogenesis of the PSI complex, but not for the production of thylakoids (Zhang et al., 2014). However, VIPP1 of *Chlamydomonas reinhardtii* is needed for the biogenesis and/or assembly of thylakoid membrane core complexes, including PSI and PSII (Nordhues et al., 2012). VIPP1 orthologs also play a key role in the exchange of proteins and/or lipids between internal membranes in cyanobacteria and chloroplasts (Hennig et al., 2015; Heidrich et al., 2017).

As mentioned above, *VIPP1* has been extensively studied in Arabidopsis but its function is scarcely known in other plants. We present in this work the molecular cloning and characterization of the tomato *white lethal seedling*-*2372* (*wls-2372*) mutant, which carries a T-DNA insertion in the *SlVIPP1* gene that causes in homozygosis early lethality. Our work reveals the requirement of tomato VIPP1 for plant survival, and its functional conservation with Arabidopsis VIPP1 supports its involvement in thylakoid membrane formation and/or maintenance.

## Materials and Methods

### Plant Material and Growth Conditions

The *wls-2372* mutant line was isolated from a T-DNA insertional collection, as described previously in Pérez-Martín et al. (2017). The TG1 line was self-pollinated to obtain its TG2 progeny, which was grown in soil conditions (a 1:1 mixture of peat and coconut fiber and, after sowing, a cover of vermiculite). Homozygous *wls-2372* mutant plants did not reach the reproductive stage so the *wls-2372* line was perpetuated by self-pollination of heterozygous plants grown in soil under greenhouse conditions, using standard practices with regular addition of fertilizers. Seedlings analyzed in this work were grown under standard conditions (16-h light/8-h darkness, ≈25°C, and 60% relative humidity) in a growth chamber. Co-segregation analysis of the mutant phenotype with kanamycin resistance conferred by the *NEOMYCIN PHOSPHOTRANSFERASE II* (*NPTII*) marker gene was carried out by sowing seeds on Murashige and Skoog (MS) agar medium supplemented with sucrose (10 g^l−1^) and kanamycin (100 mg^l−1^).

### Chlorophyll Measurement and Microscopy Analysis of Chloroplasts

Chlorophylls were extracted following the method described by Lichtenthaler and Wellburn (1983). Chlorophyll content was calculated as chlorophyll micrograms per fresh weight grams.

For chloroplast observation in living plant tissues, wild-type and mutant cotyledons at the same development stage were cut into tissue pieces of 1 cm length × 1 cm width, and they were examined under a DM2000 LED bright field microscope (Leica) equipped with a DFC420 digital camera (Leica). Confocal laser scanning microscopy images were obtained using an Eclipse Ti-E (Nikon) inverted microscope selecting the 650- to 720-nm filter. Images were captured with a C2plus (Nikon) camera using the Nikon NIS-Elements C software.

For transmission electron microscopy (TEM) analysis, tissue samples of 2 mm length × 2 mm width were fixed in a solution of 2.5% (v/v) glutaraldehyde and 2% paraformaldehyde in a 0.05 M cacodylate buffer. After fixation, samples were rinsed three times with a 0.1 M cacodylate buffer solution and further fixed with 1% osmium tetroxide for 1 h. Cotyledon tissues were then dehydrated through a series of grade ethyl alcohols ranging from 50% to 100%, and embedded and polymerized in Epon 812 resin. Resin blocks were initially thick-sectioned at 70–90 nm on an Ultracut R (Leica) ultramicrotome. After drying, sections were stained with uranyl acetate and lead citrate for contrast; later, they were viewed and pictures were taken using a Libra 120Plus (Zeiss) electron microscope.

### DNA Isolation, DNA-Blot Hybridization, Cloning of T-DNA Flanking Sequences, and PCR Genotyping

DNA isolation and DNA-blot hybridization were performed as described in Garcia-Alcazar et al. (2017). The cloning of genomic sequences flanking the *wls-2372* T-DNA insertion was performed with an anchor-PCR assay as previously described (Garcia-Alcazar et al., 2017). Primers used for this purpose are detailed in **Supplementary Table 1**. PCR amplification products obtained with this assay were purified using a GenElute PCR Clean-up Kit (Sigma Aldrich) and sequencing reactions and electrophoreses were carried out with ABI PRISM BigDye Terminator Cycle Sequencing kits on an ABI PRISM 3500 Genetic Analyzer (Applied Biosystems), respectively. The cloned sequences were compared with the SGN Database (https://solgenomics.net) to map the T-DNA insertion site to the tomato genome.

The co-segregation analysis was done by PCR amplification using the VIPP1-F1 and VIPP1-R1 primer pair (to amplify the wild-type allele), and with the VIPP1-F1 and T-DNA-LB T-DNA specific primer pair (to amplify the mutant allele). Sequences of these primers are listed in **Supplementary Table 1**. PCR was performed using 10 ng of total DNA, 50 ng of each primer, 0.25 mM dNTPs, 2.5 mM MgCl_2_, and 1 U BIOTAQTM DNA Polymerase (Bioline) in 1× Taq buffer, with the following thermal cycling conditions: 94°C for 5 min, 35 cycles at 94°C for 15 s, 60°C for 15 s, and 72°C for 2 min, and a final extension step of 5 min at 72°C. The amplification products were analyzed in 1% agarose gels in 1× SB buffer (200 mM NaOH, 750 mM boric acid, pH 8.3) and visualized with SafeView. In addition, to amplify the genomic region located between the T-DNA flanking regions, a Long-PCR assay was carried out using Elongase^®^ Enzyme Mix (Invitrogen) following the instructions given by the manufacturer.

### RNA Isolation, Reverse Transcription, and RT-qPCR Analysis

Total RNA was isolated from three cotyledons with the TRI RNA Isolation Reagent (Sigma-Aldrich) and treated with TURBO DNase (Ambion). Reverse-transcription and RT-qPCR analyses were performed as described in Garcia-Alcazar et al. (2017). RT-qPCR amplifications were performed on a 7300 Real-Time PCR System (Applied Biosystems) and each quantitation consisted of three biological replicates, each with three technical replicates (except for the RNAi lines, which consisted of a single biological replicate). The *UBIQUITINE3* housekeeping gene was used as an internal control and the absence of genomic DNA contaminating the RNA sample analyzed by RT-PCR was tested as previously described (Gimenez et al., 2010). Statistical analyses were performed using Student’s *t*-tests.

### Gene Constructions and Transgenic Lines

To silence the *VIPP1* gene, an interference RNA (RNAi) construct was generated as described in Perez-Martin et al. (2018), with some modifications. A 326-bp fragment of *Solyc11g008990* cDNA was amplified using the VIPP1-RNAi_F/R primers and cloned in sense and antisense orientations into the pKannibal vector, as described in Wesley et al. (2001). The plasmid obtained in this way was digested with *Not*I and the resulting restriction fragment was subcloned into the pART27 binary vector (Gleave, 1992).

CRISPR/Cas9 editing was performed as described in Yuste-Lisbona et al. (2020). To design the VIPP1 gRNA target sequence, we used the Breaking-Cas web software (https://bioinfogp.cnb.csic.es/tools/breakingcas/) (Oliveros et al., 2016).

To obtain the *35S_pro_ : SlVIPP1* overexpression construct, the *VIPP1* complete open reading frame was PCR amplified from tomato cv. Moneymaker cDNA using the 35S_VIPP1_F/R primer pair, to introduce a *Bam*HI restriction site (GGATCC) upstream the initiation codon and a *Kpn*I restriction site (GGTACC) downstream the stop codon (**Supplementary Table 1**). The PCR product was cloned into the pGEM-T vector (Promega), and sequenced to confirm the absence of mutations in the amplicon. The selected plasmid was digested with *Bam*HI and *Kpn*I, and the *VIPP1* cDNA was subcloned under the control of the constitutive CaMV promoter together with a plant kanamycin resistance selection marker into the pROKII binary vector, to generate an overexpression gene construct.

Overexpression and silencing binary vectors, as well as CRISPR/Cas9 constructs, were electroporated into *Agrobacterium tumefaciens* LBA4404 and Agrobacterium-mediated transformation of Moneymaker tomato cultivar cotyledons was performed as previously described (Ellul et al., 2003). In this work, eight *SlVIPP1 RNAi*, six *CR-slvipp1* (CRISPR/Cas9) and two *35S_pro_ : SlVIPP1* TG1 independent lines were characterized. The TG1 CRISPR/Cas9 lines were genotyped by PCR with primers covering the target recognition region of the gRNA (**Supplementary Table 1**). PCR products were analyzed using the Tracking of Indels by DEcomposition (TIDE) tool (https://tide.deskgen.com/), which quantifies editing efficiency and identifies the predominant types of DNA insertions and deletions (Brinkman et al., 2014). In addition, the PCR products were purified and cloned into the pGEM-T vector (Promega). At least 16 clones of each PCR product were sequenced to characterize the edited alleles in the several TG1 plants.

### Bioinformatic Analysis

Sequences of VIPP1 and PspA proteins were obtained from UniprotKB (https://www.uniprot.org/) and Phytozome v12.1 (https://phytozome.jgi.doe.gov/pz/portal.html) databases, or using BLASTP (https://blast.ncbi.nlm.nih.gov/Blast.cgi; Altschul et al., 1997), with default settings. Multiple amino acid sequence alignments were obtained using the ClustalW algorithm (http://www.ebi.ac.uk/Tools/msa/clustalo/; Larkin et al., 2007), and shaded with BOXSHADE3.21 (http://www.ch.embnet.org/software/BOX_form.html).

A phylogenetic tree of VIPP1/PspA homologs was constructed using MEGA 7.0 (http://www.megasoftware.net; Kumar et al., 2016) by the neighbor-joining method with 1,000 replications.

Predictions of natively disordered regions in Arabidopsis and tomato VIPP1 proteins were obtained at the Protein DisOrder prediction System server (PrDOS; http://prdos.hgc.jp/; Ishida and Kinoshita, 2007) with the false positive rate option set at 5%.

## Results

### The *wls-2372* Mutant Line Carries a T-DNA Insertion in the *Solyc11g008990* Gene

**Figure 1:**
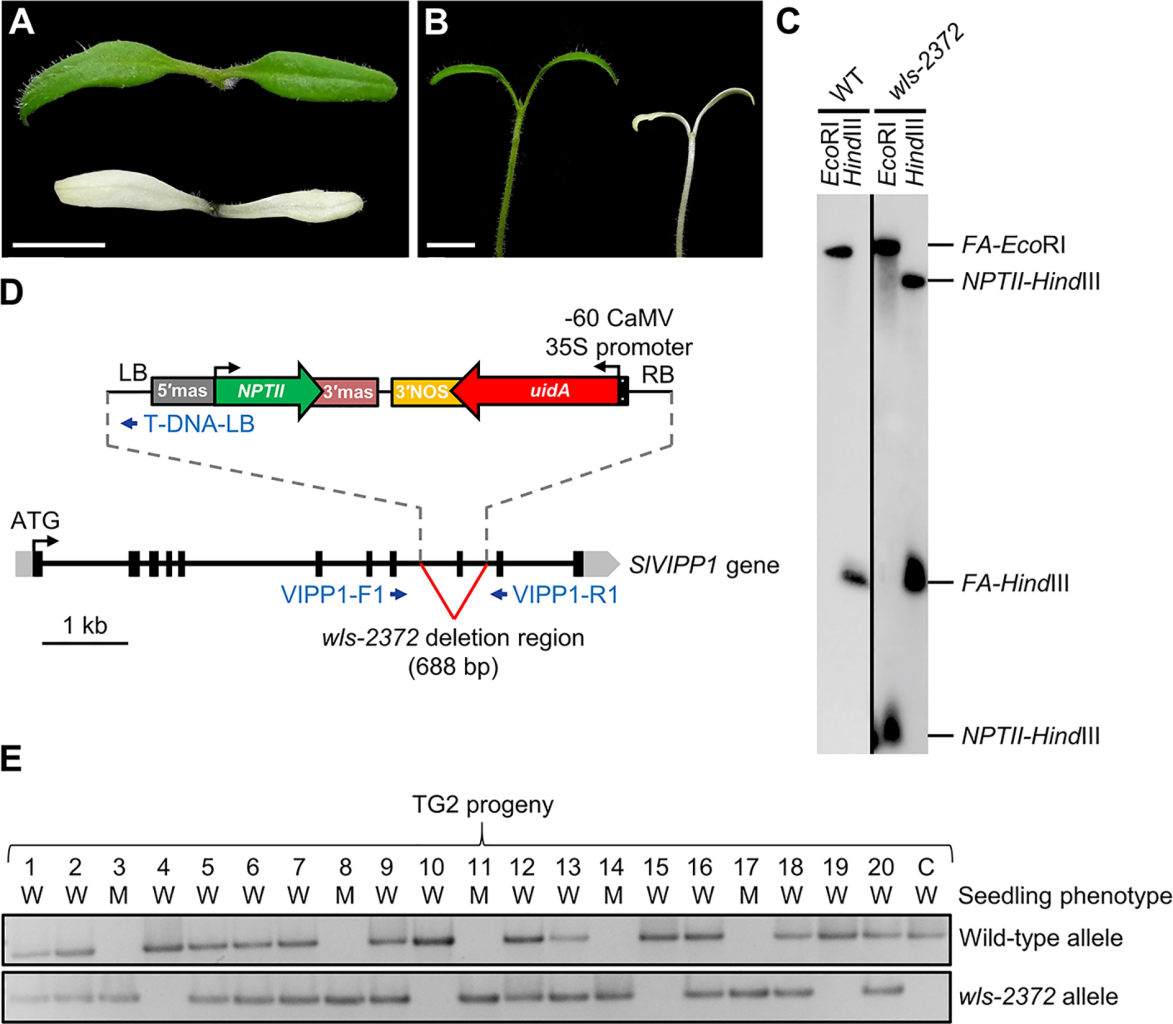
Seedling phenotype of the *wls-2372* mutant and molecular identification of the *slvipp1* mutation. **(A, B)** Pictures of one-week-old wild-type and *wls-2372* seedlings, viewed from above **(A)** and from the side **(B)**. Plants were grown in medium supplemented with 1% sucrose. Scale bars: 1 cm. **(C)** Southern blot of genomic DNA from wild-type (WT) and *wls-2372* TG1 plants, hybridized with the FA‐NPTII probe. DNA was extracted and restricted with the *Eco*RI or *Hind*III enzymes. **(D)** Structure and position of the T-DNA insertion disrupting the *SlVIPP1* gene in the *wls-2372* line. Grey boxes, black boxes and lines between boxes represent 5′- and 3′-UTRs, exons, and introns, respectively. Blue arrows represent the VIPP1-F1, VIPP1-R1 and T-DNA-LB PCR primers, which were not drawn to scale. The ↱ and ↰ symbols indicate translation start sites. **(E)** Co-segregation of the *wls-2372* mutant allele and the *wls-2372* mutant phenotype in a TG2 family. Lane numbers identify individual plants analyzed. The C (control) lane shows the amplification of wild-type genomic DNA. W and M: phenotypically wild-type and mutant seedlings, respectively.

In a large-scale screen for enhancer trap insertions (Pérez-Martín et al., 2017), we found the *wls-2372* line, some plants of which developed small whitish cotyledons when seeds were sown on Petri dishes containing agar medium supplemented with 1% sucrose (**Figures 1A, B**). Albino seedlings of the *wls-2372* line failed to develop true leaves under these *in vitro* conditions, dying few days after the emergence of their cotyledons, which suggested the existence of defects in chloroplast structure and/or function. Such albino mutant phenotype segregated in the progeny of phenotypically wild-type *wls-2372* plants in a 3:1 (wild-type:albino) ratio, indicating the inheritance of a monogenic recessive trait from a selfed heterozygous plant. This result was supported by Southern blot analysis, which indicated the presence of a single T-DNA insertion, in homozygosis, in the genome of the albino plants (**Figure 1C**). Further, seeds collected from a phenotypically wild-type plant of the *wls-2372* line were sown in culture media supplemented with kanamycin. Their phenotype characterization showed 14 Kan^S^ seedlings that died after producing green cotyledons, 28 Kan^R^ apparently wild-type seedlings that grew normally, and 6 Kan^R^ seedlings that only developed albino cotyledons and died, segregating as expected (3 Kan^R^:1 Kan^S^; chi-square = 0.444; *P* < 0.001). Similarly, the phenotypic segregation fitted a 1:2:1 (wild-type Kan^S^: wild-type Kan^R^: mutant Kan^R^; chi-square = 4.000; *P* < 0.05) ratio, although the number of lethal seedlings was half than the expected one, suggesting that lethality affected some homozygous mutant embryos.

The genomic region flanking the T-DNA insertion in the *wls-2372* line was identified by Sanger sequencing of anchor-PCR amplified products (see *Materials and Methods*). The left and right borders (LB and RB) of the T-DNA were found in the 8^th^ and 9^th^ introns of the *Solyc11g008990* gene, respectively. Therefore, the T-DNA insertion found in *Solyc11g008990* causes a deletion of 688 bp, which includes the partial loss of its 8^th^ and 9^th^ introns and the complete loss of its 9^th^ exon (**Figure 1D**). These results strongly suggest that *wls-2372* mutant plants bear a null allele of the *Solyc11g008990* gene.

The genomic organization of *Solyc11g008990* includes 11 exons, and it encodes a protein of 330 amino acids highly homologous to Arabidopsis VIPP1, which is described in the Solanaceae Genomics Network database (https://solgenomics.net/) as “Phage shock protein A, PspA”. In other databases, including The Arabidopsis Information Resource (TAIR; https://www.arabidopsis.org/index.jsp), *Solyc11g008990* is considered the tomato ortholog of Arabidopsis *VIPP1*. BLASTP analyses of the VIPP1 proteins of tomato, Arabidopsis and pea, concluded that tomato VIPP1 is 71% and 74% identical to Arabidopsis VIPP1 and pea IM30, respectively. Therefore, we will refer hereafter to *Solyc11g008990* as the *Solanum lycopersicum VIPP1* (*SlVIPP1*) gene.

To establish a possible correlation between the T-DNA inserted in the *SlVIPP1* gene and the albino phenotype of the *wls-2372* mutant, a co-segregation PCR analysis was performed using genomic DNA extracted from 20 seedlings obtained from a sibling population segregating for the mutant phenotype. The specific genomic forward (VIPP1-F1) and reverse (VIPP1-R1) primers, whose sequences hybridize at the flanking regions of the T-DNA insertion in *Solyc11g008990* (**Figure 1D**), were used to amplify the wild-type allele; VIPP1-F1 and the specific T-DNA border primer (T-DNA-LB), which hybridizes at the LB of T-DNA (**Figure 1D**), were used to amplify the *wls-2372* mutant allele. Co-segregation analysis showed that phenotypically wild-type plants were azygous or hemizygous for the T-DNA insertion, whereas all mutant plants carried the T-DNA insertion in the homozygous state (**Figure 1E**), thus indicating that the albino phenotype co-segregates with the T-DNA insertion in the *SlVIPP1* gene. Therefore, the tomato *wls-2372* insertional mutant was renamed as *slvipp1* in accordance to the name of the gene tagged in this mutant.

### Characterization of a Mutant Allelic Series of the *SlVIPP1* Gene

**Figure 2:**
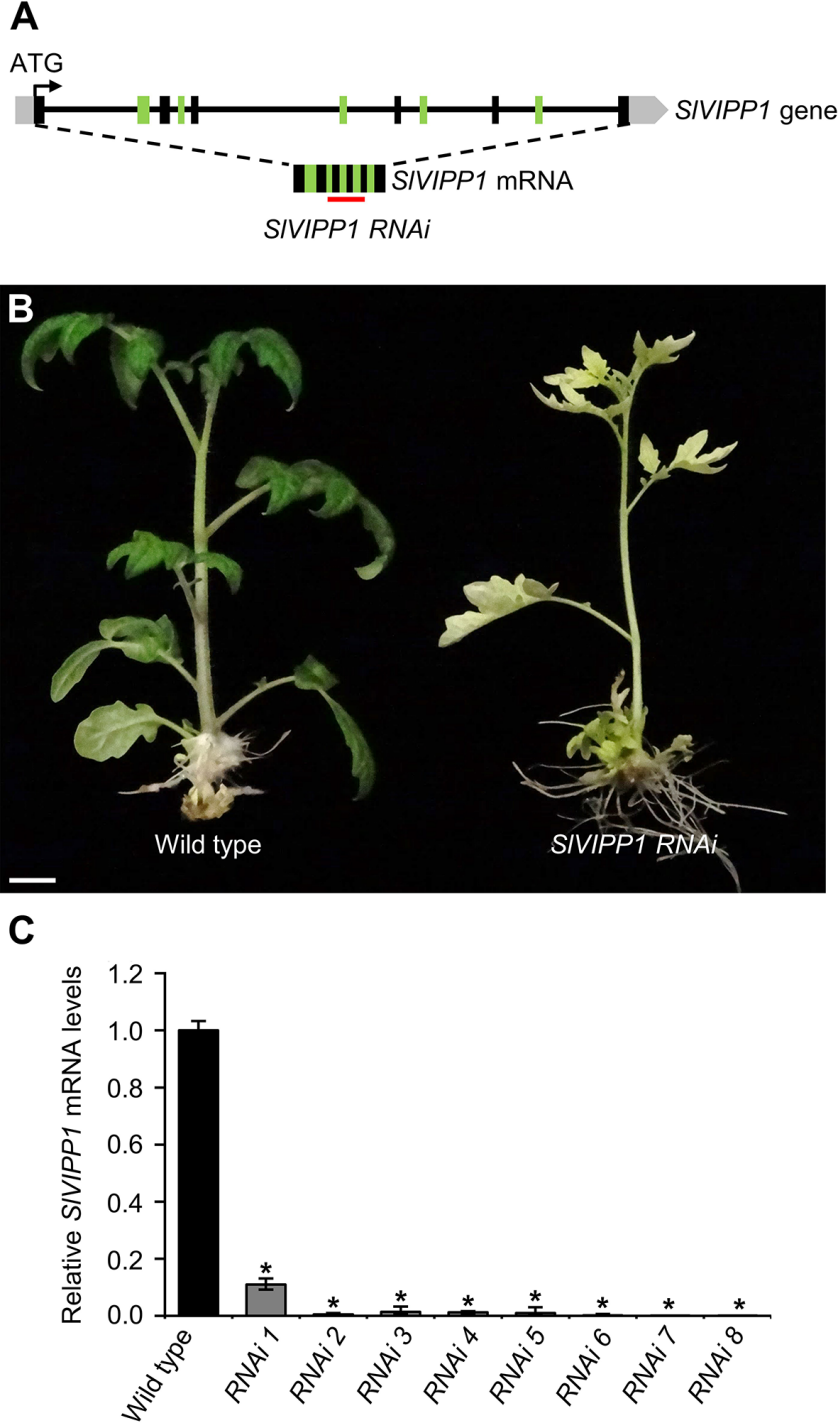
Effects of *SlVIPP1* gene silencing by RNA interference (RNAi). **(A)** Schematic representation of the *SlVIPP1* gene, as shown in **Figure 1D**, with indication of its mRNA and the RNAi used in this work. Black and green boxes indicate exons, grey boxes are 5′- and 3′-UTRs, and lines between boxes represent introns. The red line below the mRNA indicates the target sequence of RNAi. **(B)** Phenotype of RNAi transformants, compared to wild‐type plants. Pictures were taken two months after regeneration and the plants were grown in medium supplemented with 5% sucrose. Scale bar: 1 cm. **(C)** Quantitative RT‐PCR analysis of *SlVIPP1* expression in wild-type plants and eight independent RNAi lines. Asterisks indicate significant differences between the wild-type and RNAi plants in a Student’s t-test (*P < 0.001).

To confirm the molecular identity of the gene causing the albino phenotype of the *slvipp1* mutant, several RNA interference (RNAi) lines were generated to silence *SlVIPP1* through stable transformation with a gene construct designed to hybridize to a region of 326 nucleotides of *SlVIPP1* mRNA (**Figure 2A**). All TG1 plants obtained phenocopied the albino phenotype of *slvipp1* mutant plants. Nevertheless, RNAi lines grown in culture medium supplemented with 5% sucrose were able to develop 2–3 true leaves, although the seedlings eventually died without flowering (**Figure 2B**). Then, expression of *SlVIPP1* was analyzed by qRT-PCR in eight independent TG1 RNAi lines, which resulted in the detection of very low *SlVIPP1* mRNA levels in all of them, and demonstrated the efficiency of RNAi-mediated *SlVIPP1* silencing (**Figure 2C**). These results clearly show that loss of function of *SlVIPP1* causes an extremely albino phenotype and postembryonic lethality in tomato, similar to that caused by the lack of function of its ortholog in Arabidopsis (Zhang et al., 2012).

**Figure 3:**
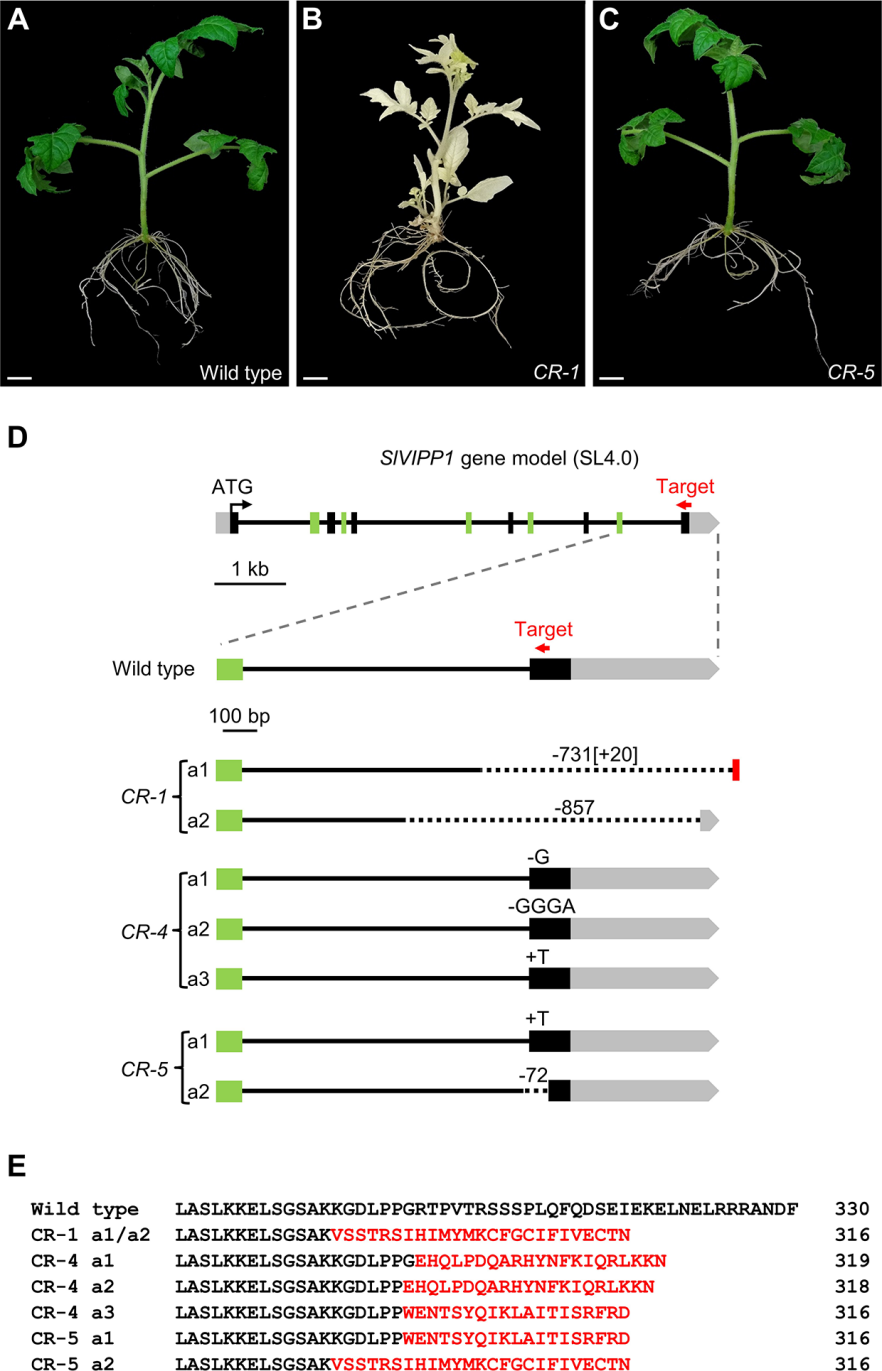
Mutant phenotypes and predicted effects on the mutated CR-SlVIPP1 proteins caused by *CR-slvipp1* alleles. **(A–C)** Phenotype of CRISPR/Cas9 transformants (*CR*) compared to wild‐type plants. Pictures were taken two months after regeneration, and the plants were grown in medium supplemented with 5% sucrose. Scale bars: 1 cm. **(D)** Schematic representation of the *SlVIPP1* gene, as shown in **Figure 2A**, and the alleles generated by CRISPR/Cas9 mutagenesis, performed using a gRNA designed to target exon 11 (red arrow) which is not drawn to scale. We identified 7 alleles (*CR-1* a1 to *CR-5* a2). The numbers preceded by minus (−) or plus (+) symbols indicate the length of large deletions (dotted lines in *CR-1* a1, *CR-1* a2, and *CR-5* a2) or insertions (red box in *CR-1* a1) found. Short deletions are indicated by a minus symbol followed by the nucleotides lost by the mutant allele. **(E)** Alignment of the C-terminal region of the wild-type SlVIPP1 protein with the predicted CR-SlVIPP1 mutated proteins obtained in this work. Numbers indicate residue positions. Mutated amino acids are shown in red.

To gain insights into the functional role of *SlVIPP1*, additional *slvipp1* mutant alleles, some of which could be hypomorphic, were obtained by using a CRISPR/Cas9 mutagenesis. With this aim, a transgene producing a guide RNA (gRNA) targeting the last exon of *SlVIPP1* (**Figure 3D**) was designed. This gRNA should edit the gene region corresponding to the C-terminal tail of SlVIPP1 protein, which is present in plants and other photosynthetic organisms but absent in non-photosynthetic bacteria (**Supplementary Figure 1**). As we already obtained insertional null alleles of the *SlVIPP1* gene, as well as transgenic RNAi lines whose lethal phenotype was the expected one for severe hypomorphic alleles, we set out to obtain some viable, weak hypomorphic alleles using the CRISPR/Cas9 gene editing technology. One gRNA, complementary to the *SlVIPP1* sequence encoding the C-terminal region of its protein product was designed. Six TG1 CRISPR lines were obtained: one of them, named as *CR-1*, only developed albino leaves when 5% sucrose was supplied to the culture medium, a phenotype that was quite similar to that of *slvipp1* mutant plants, while the remaining CRISPR lines displayed normal leaf development (**Figures 3A–C**).

The target genomic region was sequenced in the six CRISPR/Cas9 lines, finding that in three of them, i.e., *CR-1, CR-4*, and *CR-5*, the *SlVIPP1* gene had been mutated (**Figure 3D**). The *CR-1* line carried two mutant alleles (a1 and a2), both damaged in the region targeted by the gRNA. *CR-1* a1 harbored a 732-bp deletion eliminating the last 139 bp of the 10^th^ intron and the whole 11^th^ exon (the later including 120 bp translated sequence and the 3′-UTR, of 433 bp), as well as of 40 bp of the intergenic region, and incorporates an insertion of 20 bp. The *CR-1* a2 allele also contained a large deletion of 856 bp in the same region, which eliminated 359 bp of the 10^th^ intron, the whole 11^th^ exon and 377 bp of the 3′-UTR (**Figure 3D**). As a consequence, the CR-SlVIPP1-1 mutant proteins encoded by this biallelic line are predicted to be truncated and probably non-functional (**Figure 3E**). Similar large deletions have been previously described in tomato (Rodriguez-Leal et al., 2017) and mice (Shin et al., 2017; Adikusuma et al., 2018), so they seem not to be and exception among the mutations induced by the CRISPR/Cas9 system. These results fully agreed with the extreme albino phenotype exhibited by the *CR-1* line (**Figure 3B**).

Edited plants with wild-type phenotype harbored smaller mutations, all of them affecting the 11^th^ exon. Three edited alleles were identified in the *CR-4* line (a1, a2, and a3), the first and second of which harbor a single G and a GGGA deletions, respectively, while the third allele presents a single T insertion (**Figure 3D**). In the *CR-5* line, two alleles (a1 and a2) were found, one of which bear a single T insertion, and the other a deletion of 72 bp that encompasses 12 bp of the 10^th^ intron and 60 bp of the 11^th^ exon (**Figure 3D**). The open reading frames of *SlVIPP1* mRNA in the five edited alleles identified in the *CR-4* and *CR-5* lines are altered, and they are predicted to be translated into truncated proteins (**Figure 3E**). However, the *CR-4* and *CR-5* lines showed a wild-type phenotype indicating that the CRISPR/Cas9-induced mutations that they carried do not alter the functionality of the SlVIPP1 protein. In summary, the two alleles identified in *CR-1* and the a2 allele of the *CR-5* line are most likely null alleles, while at least one of the edited alleles detected in *CR-4* (a1, a2, or a3), or the a1 allele of the *CR-5* line cannot be considered as knock-out mutant alleles.

Finally, to complete the allelic series of *SlVIPP1*, transgenic plants carrying a *35S_pro_ : SlVIPP1* construct were obtained with the aim to determine the effect of *SlVIPP1* constitutive expression on tomato plant growth. Although the over-expression of *SlVIPP1* was confirmed by RT-qPCR (**Supplementary Figure 2A**), no visible phenotypic effects were observed in *35S_pro_ : SlVIPP1* transgenic plants, nor in the chlorophyll content (**Supplementary Figure 2B**) which agree to that seen when *VIPP1* was overexpressed in Arabidopsis (Zhang et al., 2016).

### The *slvipp1* Mutant Exhibits Reduced Chlorophyll Content and Dramatically Altered Chloroplast Structure

**Figure 4:**
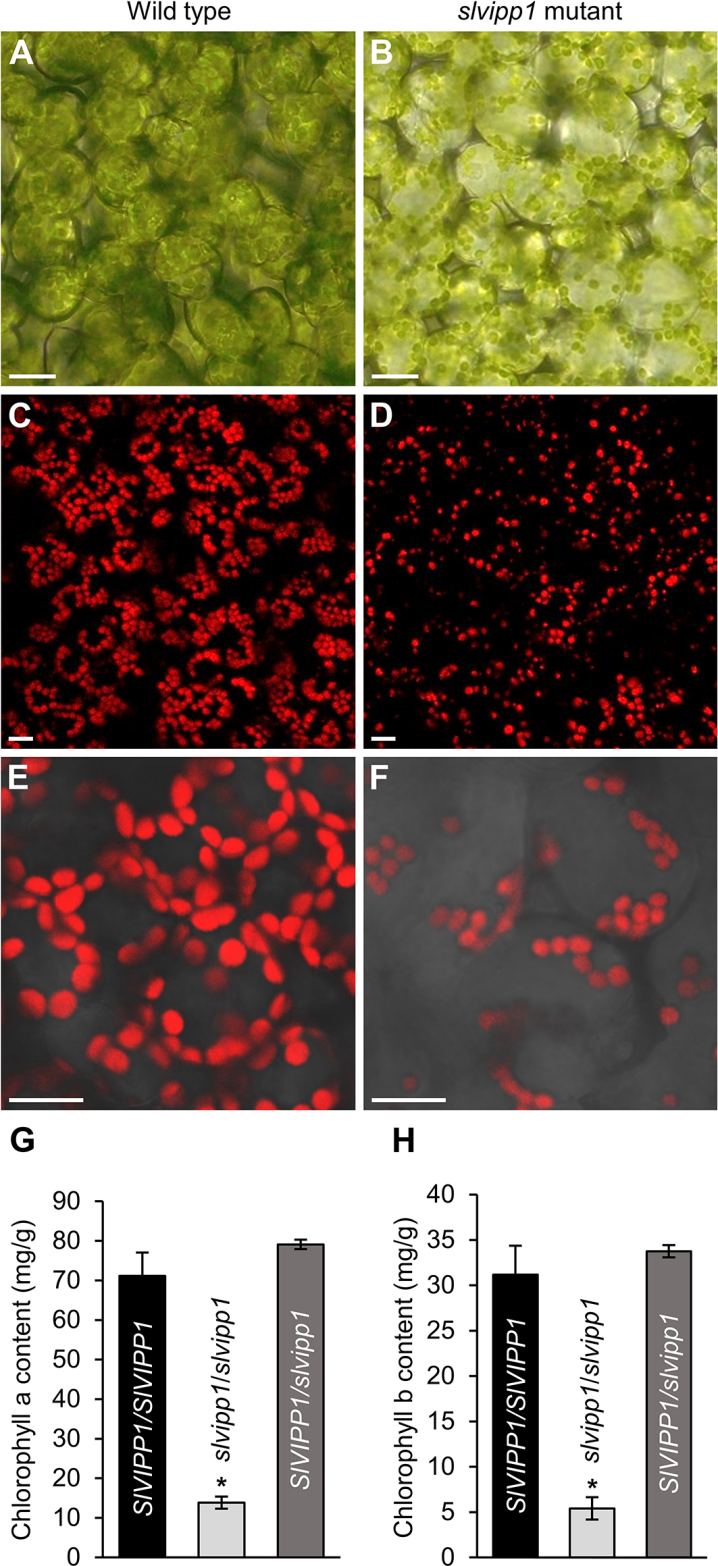
Phenotypic characterization of *slvipp1* chloroplasts. **(A–F)** Chloroplasts of wild-type and *slvipp1* mutant seedling cotyledons, examined by bright-field **(A, B)**, and confocal **(C–F)** microscopy. Chloroplasts are shown from wild-type **(A, C, E)** and *slvipp1* **(B, D, F)** plants. **(E, F)** are magnifications of **(C, D)**, respectively. Scale bars: 1 mm. **(G, H)** Chlorophyll a **(G)** and b **(H)** content in azygous (*SlVIPP1*/*SlVIPP1*) and heterozygous (*SlVIPP1*/*slvipp1*) wild-type, and homozygous *slvipp1* (*slvipp1*/*slvipp1*) seedlings. Asterisks indicate values significantly different from wild-type in a Student’s *t*-test (*P < 0.001).

Chlorosis and albinism are associated to abnormal chloroplast development and/or function. Therefore, we wondered whether *slvipp1* mutation affects chloroplast structure and chlorophyll biosynthesis. To test this hypothesis, mutant and wild-type seedling cotyledons were examined by bright-field and confocal microscopy. A reduced density and size of chloroplasts in the subepidermal tissue layer of mutant cotyledons was found as compared with wild-type ones (**Figures 4A–F**). In addition, measurements of the levels of chlorophyll a and b in cotyledons indicated that their concentrations were strongly reduced in homozygous *slvipp1* seedlings (13.85 ± 1.2 mg of pigment per g of tissue for chlorophyll a, and 5.41 ± 1.3 for chlorophyll b) compared with azygous wild-type (71.13 ± 5.91 for chlorophyll a, and 31.17 ± 3.19 for chlorophyll b) as shown in **Figures 4G, H**. Such reduction was not observed in heterozygous sibling plants, which are phenotypically wild-type (**Figures 4G, H**). These results indicate that the albino phenotype of *slvipp1* is caused by a severe chlorophyll depletion that could be the consequence of a deficit in photosynthetic activity produced by aberrant chloroplast development.

**Figure 5:**
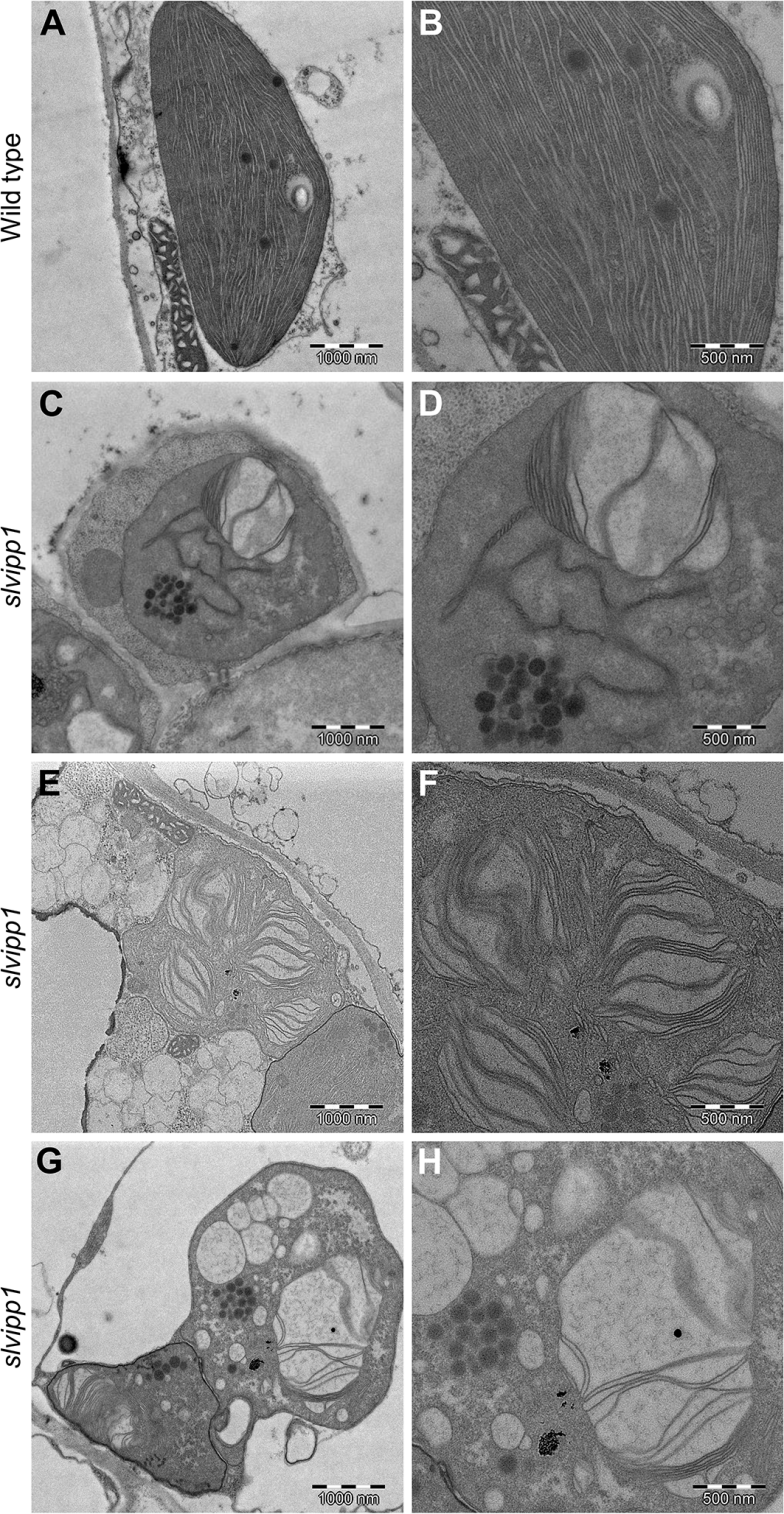
Ultrastructure of *slvipp1* chloroplasts. **(A–H)** Chloroplasts of wild-type **(A, B)** and *slvipp1* **(C–H)** seedling cotyledons, examined by transmission electron microscopy. **(B, D, F, H)** are magnifications of **(A, C, E, G)**, respectively. Scale bars: **(A, C, E, G)** 1,000 nm and **(B, D, F, H)** 500 nm.

The characteristic discoidal shape of chloroplasts and its internal architecture is well defined by three membranes. The membranous sacks known as thylakoids are mostly arranged in stacks known as grana, each containing 10–20 thylakoids. To determine if chloroplast ultrastructure is defective in *slvipp1* plants, we used transmission electron microscopy (TEM). Most chloroplasts from wild-type cotyledon cells were occupied by thylakoids, including stroma and grana, with one or two starch grains and several isolated plastoglobules associated to thylakoids (**Figures 5A, B**). Concomitant with the reduced chlorophyll levels exhibited by *slvipp1* seedlings, the ultrastructure of their chloroplasts was strongly altered, with extra stromal space and an almost complete absence of recognizable thylakoids arranged in stacked grana (**Figures 5C–H**). In addition, large vacuolated membrane structures and a high number of grouped plastoglobules were detected in mutant seedlings (**Figures 5C–H**).

### Sucrose Supply Partially Rescues the *slvipp1* Mutant Phenotype

**Figure 6:**
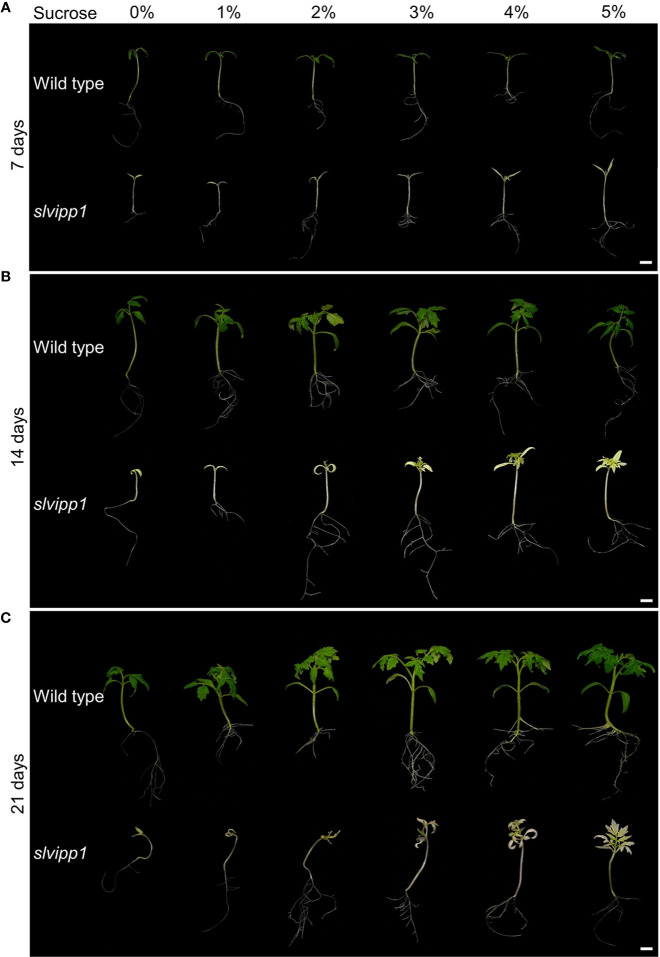
Response to sucrose of the *slvipp1* mutant. Wild-type and mutant seeds were germinated and grown on MS medium supplemented with different concentrations of sucrose (0, 1, 2, 3, 4 and 5%) at 22°C for 7 **(A)**, 14 **(B)**, and 21 **(C)** days. Scale bars: 1 cm.

Using light-dependent reactions, chloroplasts produce energy-storing monosaccharides, which are then used for the biosynthesis of polysaccharides in the cytosol, one of which is sucrose, which in turn are essentially required for cell functions. Some mutants affected in genes encoding proteins involved in chloroplast development display severe abnormalities and an albino phenotype, which can be partially rescued by supplementing the culture medium with sucrose (Gerdes et al., 2006; Teng et al., 2006; Myouga et al., 2013). To determine if this was the case for the *slvipp1* mutant, seeds were sown in Murashige and Skoog (MS) media supplemented with different concentrations of sucrose (from 1% to 5%). Seven days after sowing (das), significant differences were not observed in the growth rate between wild-type and mutant seedlings, the pigmentation of the cotyledons being the only exception (**Figure 6**). However, *slvipp1* mutant seedlings grown on media with at least 3% sucrose showed whitish true leaves 14 das, with an otherwise normal developmental pattern; this phenotype greatly differed from that observed at lower sucrose concentrations. Mutant seedlings continued their growth until 21 das, though more etiolated than the wild-type, proving that the vegetative abnormalities caused by *slvipp1* mutation were partially rescued depending upon sucrose concentration of the medium. However, mutant seedlings eventually died without flowering after 44 das, most likely due to depletion of the photoassimilate source and photosynthesis inhibition. These results allow us to corroborate that lack of *SlVIPP1* function severely impairs photosynthesis, making the mutant plants to require an external supply of carbohydrates for vegetative development.

### Evolutionary Conservation of VIPP1 Homologs

Given the crucial function that the Arabidopsis and tomato *VIPP1* genes seem to have in plant development, we wondered about the grade of conservation of VIPP1 orthologs from the major angiosperm lineages (Myburg et al., 2014). High conservation was found among these proteins, which was lesser for the 70 N-terminal amino acids (**Supplementary Figure 3**). The N-terminal regions are likely to correspond to the transit peptide, which is required for its import into the chloroplast, but that is not present in the mature protein, and it is not usually conserved. Therefore, the similarity among VIPP1 mature proteins was determined using the ChloroP Server, which predicts the presence of chloroplast transit peptides (http://www.cbs.dtu.dk/services/ChloroP/). The predicted number of amino acids of the chloroplast transit peptide of tomato VIPP1 was 58, 64 for Arabidopsis, and 43 for pea. These values are in agreement with those already published for Arabidopsis and pea, in the UniprotKB database (https://www.uniprot.org/). Moreover, BLASTP analysis of the predicted mature proteins showed that identity reached 79.4% between tomato and Arabidopsis, and 80.2% for tomato and pea, respectively. These results revealed a high degree of conservation among the VIPP1 mature proteins of higher plants. The high conservation among plant VIPP1 mature proteins includes their C-tail region, which in Arabidopsis and tomato extends from residues 260 and 280, respectively. Strikingly, a short region comprissing residues 304-309 of Arabidopsis VIPP1 and 303-309 of SlVIPP1 was not conserved, although its biological significance is not known so far (**Supplementary Figures 1** and **3**).

The region between residues 289 and 323 of the C-terminal tail in Arabidopsis, which is intrinsically disordered, is required for maintaining membrane integrity in stressful conditions (Zhang et al., 2016). Intrinsically disordered regions are segments that lack a unique three-dimensional structure because they do not contain sufficient hydrophobic amino acids to mediate co-operative folding (Babu, 2016). They generally show a variety of conformations that are in dynamic equilibrium under physiological conditions. The PrDOS tool (http://prdos.hgc.jp/cgi-bin/top.cgi) was used for searching intrinsically disordered regions in SlVIPP1. PrDOS combines results based on the local amino acid sequence and on the known structures of homologous proteins (Ishida and Kinoshita, 2007). Two regions were predicted to be disordered, between amino acids 1 to 21 and the C-terminal region of SlVIPP1 (**Supplementary Figure 4**). The first region is part of the chloroplast signal peptide, as previously mentioned, while the second matches the disordered region of the C-terminal tail of Arabidopsis VIPP1 (**Supplementary Figures 1** and **3**). In our UniprotKB database analysis, the 286-330 amino acid region of SlVIPP1 was also found predicted to be disordered.

We also obtained a phylogenetic tree of PspA and VIPP1 proteins from Bacteria, Chlorophytes, Bryophytes, and Embryophites. Some organisms harbor only one PspA (as *Escherichia coli*) or VIPP1 (as tomato) protein, others harbor both, PspA and VIPP1 proteins (as *Synechocystis*), and others have two (as *Zea mays*) or even three (as *Glycine max*) VIPP1 proteins. In the case of plants with two VIPP1 protein, both paralogs usually clustered, suggesting that are the result of a recent duplication and could be co-orthologs. However, the *Gylcine max* genome could encode one additional VIPP1-related function, because one of its three paralogs clustered in a different node (**Supplementary Figure 5**).

## Discussion

### *SlVIPP1* Function Is Essential for Chloroplast Development and Plant Survival

Molecular characterization of the tomato enhancer trap *wls-2372* line led to the isolation of the *SlVIPP1* gene, which encodes a PspA-like protein that is the most likely ortholog of Arabidopsis VIPP1. An essential role in thylakoid membrane biogenesis has been proposed for Arabidopsis VIPP1, based on the morphological, physiological, and cellular phenotypes exhibited by loss-of-function *vipp1* mutants. Arabidopsis *vipp1* knockdown mutants are albino in sugar-free growth medium and their chloroplasts do not develop functional thylakoid membranes and, in consequence, photosynthesis is abolished in these mutants (Kroll et al., 2001). Chloroplasts of tomato *slvipp1* seedlings exhibited morphological abnormalities similar to those reported for the Arabidopsis *vipp1* mutant (Zhang et al., 2012). Further characterization by electron microscopy of chloroplast ultrastructure in *slvipp1* revealed similar aberrant structures to that previously described for Arabidopsis *vipp1* chloroplasts, mainly, unrecognizable thylakoid membranes, enlarged stromal space with large vacuolated membrane structures, and high number of grouped plastoglobules. Plastoglobules are lipoprotein subcompartments attached to thylakoids, which become visible as dark round bodies after postfixation with osmium tetroxide. In greening and mature chloroplasts, most plastoglobules are single and coupled to a thylakoid membrane, and its number decreases during chloroplast biogenesis and increases in mutants impaired in thylakoid development (Mechela et al., 2019), as shown in *slvipp1* chloroplasts. According to the abnormal development of *slvipp1* chloroplasts, photosynthesis is impeded and consequently, mutant seedlings lack of chlorophylls exhibiting their characteristic albino phenotype; then, after a few days, *slvipp1* seedlings prematurely died before any true leaves were initiated. Importantly, characterization of RNAi and CRISPR/Cas9 lines proved that genetic causality of *slvipp1* mutant phenotype relies on the *SlVIPP1* gene function. Together, our results indicate that the albino phenotype and subsequent lethality of *slvipp1* mutant plants are caused by the abnormal development of thylakoid membranes, and therefore, that *SlVIPP1* is absolutely required, from the earliest developmental stages, for the development and/or maintenance of chloroplast functionality and survival in tomato plants. This conclusion is consistent with the expression pattern of the *SlVIPP1* gene, which shows the highest transcript accumulation in leaf tissues and the lowest level of expression in roots (Zouine et al., 2017).

Two different roles have been proposed for different VIPP1 orthologs. In *Synechocystis*, VIPP1 seems to play a role in photosynthetic complex biogenesis or/and assembly, since its loss of function causes the degeneration of thylakoid membranes (Westphal et al., 2001; Fuhrmann et al., 2009). Furthermore, a role has been proposed for *Chlamydomonas* VIPP1 in the biogenesis/assembly of thylakoid membrane core complexes (Nordhues et al., 2012). In Arabidopsis, plants lacking VIPP1 function showed defects in thylakoid membrane formation but not in the assembly of protein complexes in thylakoid membranes indicating that VIPP1 plays a role in thylakoid membrane maintenance (Aseeva et al., 2007). Our results did not allow us to accurately determine whether SlVIPP1 is required for biogenesis or maintenance of thylakoid membranes, or for the biogenesis or assembly of photosynthetic complexes into the thylakoid membrane. However, we consider reasonable to assume that the function of SlVIPP1 is closer to that of Arabidopsis than of cyanobacteria.

The CRISPR/Cas9 allelic series of *SlVIPP1* gene here reported not only supports the essential role of *SlVIPP1* in plant development but also provides useful information about SlVIPP1 protein domains. The six different CRISPR/Cas9 alleles characterized in this work harbored deletions that partially or totally removed the 11^th^ exon of *SlVIPP1*. However, the *CR-4* and *CR-5* lines developed normally without any morphological alterations, suggesting that the editions found in the *CR-4* a1, *CR-4* a2, *CR-4* a3, and *CR-5* a1 alleles (**Figure 3**) do not impair SlVIPP1 protein function. On the contrary, the *CR-1* line showed a strong albino phenotype caused by two mutant alleles bearing long deletions and insertions, which are likely to yield truncated and non-functional proteins. Therefore, *CR-1* a1 and *CR-1* a2 can be considered as null alleles of *SlVIPP1* gene. Similar to the *CR-1* a1 and *CR-1* a2 alleles, the 72 bp-deletion allele *CR-5* a2 predicts the same truncated form of the SlVIPP1 protein due to the lack of processing of intron 10, which generates a premature stop codon in the three respective transcripts (**Figures 3D, E**). The severe albinism of the *CR-1* line (**Figure 3B**) and the absence of phenotypic alterations in the *CR-5* line (**Figure 3C**) indicate that the *CR-5* a1 allele acts as a dominant wild-type allele, which complements the effect of the 72 bp-deletion of the *CR-5* a2 allele. Furthermore, such complementation capability may rely on the KGDLPP domain encoded by the 5′ region of exon 11^th^, which is a significant difference between both CRISPR/Cas9 alleles (**Figures 3D, E**), suggesting that this domain is essential for SlVIPP1 protein functionality. The KGDLPP domain is placed at the beginning of the C-terminal region, and it is absent in bacteria PspA proteins (**Supplementary Figure 1**), but conserved among plant VIPP1 orthologs (**Supplementary Figure 3**), where its third residue can be D (aspartate) or E (glutamate), both acidic amino acids with a negatively charged side chain (**Supplementary Figure 4**). Furthermore, it has been described that the C-terminal extension contains an α-helical structure that is connected to the rest of the protein by a random coil spacer that begins with one leucine and one or two proline residues (LP or LPP) (Zhang et al., 2016), which are the last three amino acids in the KGDLPP domain. BLASTP searches here performed with the tomato KGDLPP and the Arabidopsis KGELPP sequences in species-specific and generic protein databases revealed that this region is only present in plant VIPP1 proteins. The detailed characterization of these CRISPR/Cas9 allelic series and the further directed mutagenesis of the KGDLPP domain will provide us a better understanding of the functional relevance of the SlVIPP1 protein domains.

### Exogenous Sucrose-Supply Partially Rescue the Loss of Function of the *SlVIPP1* Gene

Sugars are the most relevant products of photosynthesis. Plant culture media are usually supplemented with a moderately low concentration of sucrose to improve growth, since plants growing under *in vitro* conditions metabolize sucrose to yield glucose, which is stored or immediately used as energy source. Hence, the seedling lethality of many chlorotic or albino mutants is suppressed by growing plants on a medium supplemented with sucrose. This is the case for the Arabidopsis *vipp1* knockdown mutants, which unlike the wild-type, are unable to grow photoautotrophically on soil but develop as pale-green plants that may complete their life cycle on sucrose-supplemented agar media (Kroll et al., 2001). The growth in the absence of sucrose of our *slvipp1* mutant was comparable to that of Arabidopsis, displaying an albino phenotype with a strongly arrested development when they grew *in vitro* without an exogenous carbon source or with low concentrations of sucrose. Under these growth conditions, plants showed arrested development, producing only two white cotyledons. However, the arrested seedling growth was partially rescued with higher concentrations of sucrose, producing *slvipp1* plants that were able to develop true leaves but unable to photosynthesize, as expected from their dramatically aberrant chloroplasts. These results indicate that SlVIPP1 protein, as its Arabidopsis VIPP1 ortholog, is required to establish photoautotrophic growth during early development, and hence, to confer photosynthetic activity to tomato seedlings.

### VIPP1 Is Highly Conserved in Plants

Along the evolution of plants, many genes of cyanobacterial origin were transferred to nuclear plant genomes, including those involved in oxygenic photosynthesis, a process that is specific of cyanobacteria and plant chloroplasts. Furthermore, many homologs of cyanobacterial proteins act within plant chloroplasts and are required for the biogenesis and function of this organelle; one such protein is VIPP1 (Westphal et al., 2001). The alignment of VIPP1 plant orthologs revealed a high degree of conservation, except for the N-terminal amino acids, which can be explained because most eukaryotic proteins with a chloroplastic function are encoded by nuclear genes and synthetized as inactive pre-proteins. Their most N-terminal amino acids conform the transit peptide, which is used for their import into the chloroplast, where it is removed. In fact, the VIPP1 ortholog of *Pisum sativum* was identified, as previously mentioned, as a 37-kDa precursor, whose processing results into a mature protein of 30 kDa (Li et al., 1994). In the UniprotKB database, the amino acids 1-44 of pea IM30 and 1-64 of Arabidopsis VIPP1 are annotated as their respective transit peptides (https://www.uniprot.org/). On the other hand, transit peptides are highly heterogeneous in length (from 20 to more than 100 amino acids) and sequence, hindering its prediction in not few cases (Jarvis and Robinson, 2004).

As previously mentioned, *Synechocystis* sp. has two homologs of SlVIPP1: the PspA and Vipp1 paralogs. *Escherichia coli*, which is not a photosynthetic organism, only has a PspA protein. Both *Escherichia coli* and *Synechocystis* sp. PspA proteins lack the C-terminal extension that is present in VIPP1 proteins from photosynthetic organisms, including tomato SlVIPP1 (Westphal et al., 2001). However, the C-terminal extension of Vipp1 of *Synechocystis* sp., *Chlamydomonas reinhardtii* and *Physcomitrella patens* presents low conservation with plant VIPP1 proteins (**Supplementary Figure 1**). Furthermore, previous studies have shown that a C-terminally truncated protein does not replace the function of a wild-type protein in *Synechocystis*, but it does in Arabidopsis (Zhang et al., 2016; Hennig et al., 2017). The CRISPR/Cas9 *CR-1* line reported in this research produces two mutant proteins lacking the wild-type C-terminal region; this line shows a fully albino phenotype. This observation suggests that, contrary to what was found in Arabidopsis, the C-terminal extension is essential for tomato plant growth. We found that the C-terminal region: of SlVIPP1 is predicted to be intrinsically disordered (**Supplementary Figure 4**) suggesting that it may play a key role in the maintenance of thylakoid membranes under stress tolerance, as shown for Arabidopsis (Zhang et al., 2016). However, there are no experimental evidences supporting this hypothesis. Future deletion analysis of this C-terminal region could be useful for determining its function, if any. This assumption can be extended to all VIPP1 plant proteins, since their highly conserved regions includes their C-terminal domains (**Supplementary Figure 3**).

## Data Availability Statement

The original contributions presented in the study are included in the article/**Supplementary Material**; further inquiries can be directed to the corresponding author.

## Author Contributions

RM-P, MG-A, CC, BP, BG-S, and AA performed field and laboratory experiments. RM-P wrote the manuscript draft, which was then edited by RL, RM-P, JC, and FY-L. VM and RL conceived the research plans, supervised the study, and collaborated in data analysis. All authors contributed to the article and approved the submitted version.

## Conflict of Interest

The authors declare that the research was conducted in the absence of any commercial or financial relationships that could be construed as a potential conflict of interest.
