# Supplementary material for: The Tomato SlVIPP1 Gene Is Required for Plant Survival Through the Proper Development of Chloroplast Thylakoid Membrane
Source: Front Plant Sci. 2020 Aug 26;11:1305. doi: 10.3389/fpls.2020.01305 (PMC7479267; doi:10.3389/fpls.2020.01305)
Supplement: Supplementary file 1 [file DataSheet_1.pdf]

Supplementary Table 1. Oligonucleotides used in this work

Purpose		Oligonucleotide name(s)	Oligonucleotide sequence (5′→3′)	
			Forward primer (F)	Reverse primer (R)
Anchor-PCR analysis		Adaptor-1	CTAATACGACTCACTATAGGC	
		Adaptor-2	CTATAGGGCTCGAGCGGC	
		Adaptor-3	AGCGGCGGGGAGGT	
		Anchor Right-1	ACAGTTTTCGCGATCCAGAC	
		Anchor Right-2	GGTCTTGCGAAGGATAGTGG	
		Anchor Right-3	CTGGCGTAATAGCGAAGAGG	
		Anchor Left-1	ATCGGTCTCAATGCAAAAGG	
		Anchor Left-2	CGTCGAAATAAAGATTTCCGAAT	
		Anchor Left-3	ATAATAACGCTGCGGACATCTAC	
Genotyping of	*SlVIPP1*	VIPP1-F1/R1	CTCAGCCTTCGAAAGAATGGAA	CATCATCAACAGAAGAGGTCTC
	slvipp1-1	VIPP1-F1/T-DNA-LB		TTGGCGTGTCAGCGTATCTA
	CRISPR	VIPP1-CR-F1/R1	TCCTTGCTTTGGCAAATCTC	GAAAACATTTGGGTGGCAGT
Construction of	*35S*$_{pro}$*:SlVIPP1*	35S_VIPP1_F/R	GGATCCGGCTACCCATTTGGCTGAA	GGTACCGGAAGATCCAAAAGCCACAA
	CRISPR	VIPP1-CRg_F/R	ATTGTAACTGGTGTTCTCCCCGG	AAACCCGGGGAGAACACCAGTTA
	RNAi	VIPP1-RNAi_F/R	TCTAGACTCGAGTGCACAACAAGCT TCTGAGG	ATCGATGGTACCCCATTCTTTCGAAGG CTGAG
qRT-PCR analysis		LE-Ubi-F/R	CCAAGATCCAGGACAAGGAA	AAATCAAACGCTGCTGGTCT
		ALQ-Pro-F/R	GGGAAGGTTCCCTACAATG	GGGGTTGCTCATCCATTA
		VIPP1-Fz/Rz	CTCAGCCTTCGAAAGAATGGAA	CATCATCAACAGAAGAGGTCTC
