# Supplementary material for: The Tomato SlVIPP1 Gene Is Required for Plant Survival Through the Proper Development of Chloroplast Thylakoid Membrane
Source: Front Plant Sci. 2020 Aug 26;11:1305. doi: 10.3389/fpls.2020.01305 (PMC7479267; doi:10.3389/fpls.2020.01305)
Supplement: Supplementary file 2 [file DataSheet_2.pdf]

```
E. coli            1 ------------------------------------------------------------
S. sp.             1 ------------------------------------------------------------
C. reinhardtii     1 -----------------------------------------------------MSL----
P. patens          1 MAGVALGLVGPGLELVASKRALCPGASFSSPFCLSCSASVSSNSSTRARSPKALVLRSSF
S. lycopersicum    1 ----------------MAVRTPITGIT--MSSA------PSISSCNRVVVVKVMPF----
A. thaliana        1 ---------------MALKASPVTGLF--PPLRPT-ASSSPSTSSNRPCSLRILPL----
consensus          1

E. coli            1 -------------------------------------------MGIFSRFADIVNANINA
S. sp.             1 -------------------------------------------MGLFDRLGRVVRANLND
C. reinhardtii     4 LASSRLNTLR---------SGQRSGVRSAVPAVRRSRKAVVVQANLFSRAARIVNSWATN
P. patens         61 VSRTTHSSFWDGGVGACVLA---LAVEDSI-KQRKRGGALCAQANIFERVVRIVRSYANA
S. lycopersicum   33 -----RASFFGQGVGAVKLAGLQLTHSYRSRCNSHGGGALGARMNLFDRFARVVKSYANA
A. thaliana       39 -----RTSFFGNSSGALRVNVLRLACDNRLRCNGHG-----ATMNLFERFSRVVKSYANA
consensus         61                                              .* *  ..*    .

E. coli           18 LLEKAEDPQKLVRLMIQEMEDTLVEVRSTSARALAEKKQLTRRIEQASAREVEWQEKAEL
S. sp.            18 LVSKAEDPEKVLEQAVIDMQEDLVQLRQAVARTIAEEKRTEQRLKQDTQEAKKWEDRAKL
C. reinhardtii    55 VVSNAEDPEKLLDQVVEEMQGDLIKMRQAAATILAQQKQIETKYKQAQTTADDWLRRAEL
P. patens        117 IVSSAEDPEKLLDQTVLEMNEDLIKMRQASAQVLASQKQLENKYKAAQTAADDWYRRAKL
S. lycopersicum   88 LISTFEDPEKILEQTVLEMNNDLIKLRQATAQVLASQKQLENKYKAAQQASEDWYRRAQL
A. thaliana       89 LISSFEDPEKILEQTVIEMNSDLTKMRQATAQVLASQKQLQNKYKAAQQSSDDWYKRAQL
consensus        121 ...  ***.*.... . .*. .*. .*.. *  .*  *..  . . .     .*  .* *

E. coli           78 ALLKEREDLARAALIEKQKLTDLIKSLEHEVTLVDDTLARMKKEIGELENKLSETRARQQ
S. sp.            78 ALSNGDENLAREALARKKSLTDTATAYEAQLAQQRTMSENLRRNLAALEAKISEAKTKKN
C. reinhardtii   115 AVQKGEDDLAKEALKRRKTYQEQADQLKVQVDQLSGASGDVLNNTRALEAKLQEARSKKE
P. patens        177 ALEKGDEDLAREALKRRKDYEESAKALKSQLDQQKGVVDKLISNTRLLESKISEAKSKKD
S. lycopersicum  148 ALGKGDEDLAREALKRKKSYADNANALKTQLDQQKAVVDNLVSNTRLLESKIQEAKSKKD
A. thaliana      149 ALAKGDEDLAREALKRRKSFADNATALKTQLDQQKGVVDNLVSNTRLLESKIQEAKAKKD
consensus        181 *. .....**..** ...   .  .  .  .. .       .  .   ** *. *.. ..

E. coli          138 ALMLRHQAANSSRDVRRQLDSGKLDEAMARFESFERRIDQMEAEAESHSF-GKQKSLDDQ
S. sp.           138 MLQARAKAAKANAELQQTLGGLGTSSATSAFERMENKVLDMEATSQAAGE-LAGFGIENQ
C. reinhardtii   175 TLKARAASAKTSQQIQEMMSGLNTSNAVVAFDKMEQKVLSMEAQAESTKMLVGSDTIDNK
P. patens        237 TLKARAQSAKTSQKVNEMLGNINTSGALAAFEKMEEKVTALEAESEALNQ-LSTDDLAAK
S. lycopersicum  208 TLKARAQSAKTATKVSEMLGNVNTSSALSAFERMEEKVLTMESQADALNQ-LTSDELEGK
A. thaliana      209 TLLARARTAKTATKVQEMIGTVNTSGALSAFEKMEEKVMAMESEADALTQ-IGTDELEGK
consensus        241  * .*.  *..   .   .  . ..  *. .*...* ..  .*   .     .    ..

E. coli          197 FAELKADDAISEQLAQLKAKMKQDNQ----------------------------------
S. sp.           197 FAQLESSSGVEDELAALKASMAGEALPGTSAATPRLEASPVDSSVPTSNASQDDAVIDQE
C. reinhardtii   235 FKQLES-GTVDDELAALKRGMLPSTTSAVGSLPEP--RA--------------VDALDLE
P. patens        296 FALLES-DSVDDDLASLKQDVLGSSK-RKGELPEG--RSQAVSSSSKTPYPFKDSEIERE
S. lycopersicum  267 FALLET-SSVDDDLASLKKELSGSA--KKGDLPPG--RTPVTR--SSSPLQFQDSEIEKE
A. thaliana      268 FQMLET-SSVDDDLADLKKELSGSS--KKGELPPG--RSTVAA---STRYPFKDSEIENE
consensus        301 *  *..   ....** **  .                                   .. .

E. coli              -----------
S. sp.           257 LDDLRRRLREI
C. reinhardtii   278 LEALRKKARAE
P. patens        352 LNELRKRANDF
S. lycopersicum  320 LNELRRRANDF
A. thaliana      320 LNELRKKANDF
consensus        361 .  ....
```

Supplementary Figure 1. Amino acid sequence alignment of VIPP1/PspA homologs from prokaryotic and eukaryotic organisms carrying out oxygenic photosynthesis.
Sequence alignment of full-length *Escherichia coli* (*E. coli*) PspA (P0AFM6), *Synechocystis* sp. (*S.* sp) PCC6803 VIPP1/IM30 (BAK511309), *Chlamydomonas reinhardtii* (*C. reinhardtii*) *VIPP1* (XP_001693830), *Physcomitrella patens* (*P. patens*) *VIPP1* (XP_024367355), tomato (*S. lycopersicum*) VIPP1 (XP_004250100) and Arabidopsis (*A. thaliana*) VIPP1 (NP_564846). Numbers indicate residue positions. The letters shaded in black or grey indicate identical and similar residues shared by at least three of the four sequences, respectively. The red and black lines under the consensus sequence correspond to the N-terminal signal peptide and the C-terminal intrinsically disordered region, respectively, as annotated in UniprotKB. The red rectangle indicates the KGDLPP domain that is absent in the CR-1 a1, CR-1 a2 and CR-5 a1 mutant proteins. The alignment shows that the N-terminal signal peptide (underlined in red) is only present in both plants and mosses, and the C-terminal intrinsically disordered region (underlined in black), is absent from the non photosynthetic bacteria (*E. coli*), but present in the photosynthetic bacteria (*S.* sp.), the single-cell green algae (*C. reinhardtii*), the mosses (*P. patens*) and the two plants.

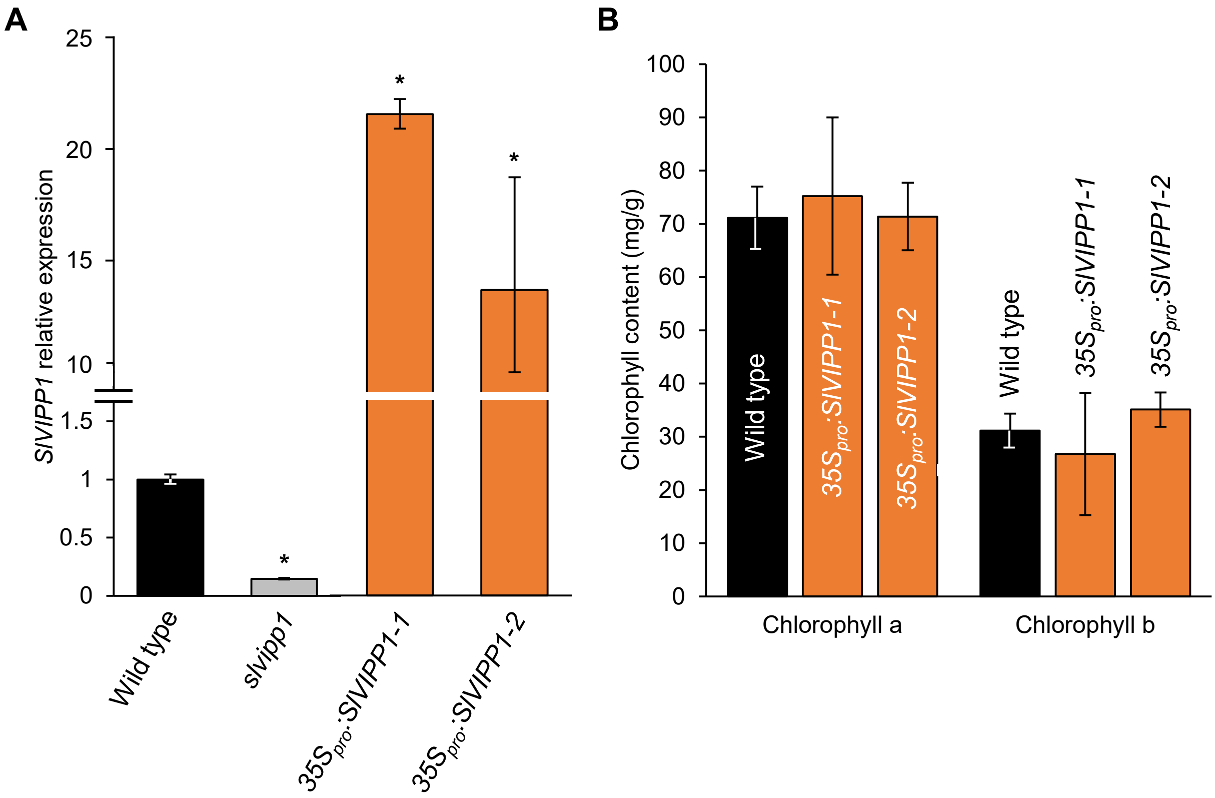

Supplementary Figure 2. Effects of *SlVIPP1* overexpression.

(A) Quantitative RT-PCR analysis of *SlVIPP1* expression in wild-type, mutant *slvipp1* and transgenic *35S*$_{pro}$*:SlVIPP1* TG2 plants derived from two independent TG1 transgenic lines (*35S*$_{pro}$*:SlVIPP1-1* and *35S*$_{pro}$*:SlVIPP1-2*). Asterisks indicate significant differences with the wild type, found in a Student's *t*-test (*P<0.001). **(B)** Chlorophyll a and b content in wild-type and *35S*$_{pro}$*:SlVIPP1* transgenic plants.

```
P. dactylifera      1 MAIKAPIG-GLRL--ASPPSASLRA--------LPKRAIVACPPLRTSFFNGGV-GALKA
O. sativa           1 MEIRAPPT-SLRL--APPPPASA---------------SFRRTALRTSFLNGSV--SLRL
A. thaliana         1 MALKASPVTGLFPPLRPTASSSPST-------SSNRPCSLRILPLRTSFFGNSS-GALRV
P. trichocarpa      1 MAIKSQLISGLTLPLPHPPSTSSSSNNNSNSNNNTILSLVKRRNLKTSFFNGGV-EALKI
G. max              1 MAAKLQIFTGLPP--A--PFQSSS-----------STLCVLKKPLTTSFFGTARVEVLKL
S. lycopersicum     1 MAVRTPIT-GITM--SSAPSISSCN----------RVVVVKVMPFRASFFGQGV-GAVKL
V. vinifera         1 MATKVPIGAGLSL--ASPPCA----------------SNVRSLVLKSAFFGHGV-GALKV
consensus           1 *...  .  .. .     .  .                 ..   ....*.  ..  ....

P. dactylifera     49 VEIRTSRFNRQRCGGLGGGGLGAQMNLFDRLGRVIKSYVNAIVSSFEDPEKILEQAVMDM
O. sativa          41 IQVRQSNVNRFKCNGIR-------SNLLDRFSRVVKSYANAVLSSFEDPEKILDQAVLEM
A. thaliana        53 NVLRLACDNRLRCNGHG-----ATMNLFERFSRVVKSYANALISSFEDPEKILEQTVIEM
P. trichocarpa     60 TRIRTGLSTRSHCYGHGGGALGTRMNLFDRFARVVKSYANAIVSSFEDPEKILEQTVLEM
G. max             46 RVMRI------AKPARGGGALGARMNLFDRFARVVKSYANAVLSSFEDPEKILEQAVLEM
S. lycopersicum    47 AGLQLTHSYRSRCNSHGGGALGARMNLFDRFARVVKSYANALISTFEDPEKILEQTVLEM
V. vinifera        42 RGIRIARSSTYRCNGHGGGALGTQMNLFDRFARVVKSYANAVLSSFEDPEKILEQTVLEM
consensus          61   ..     .  .. ........  .**..*..**.***.**..*.********.* *..*

P. dactylifera    109 NNDLTKMRQATAQVLASQKRLENKYRATQQASDDWYRRAQLALEKGDEDLAREALKRRKS
O. sativa          94 NDDLTKMRQATAQVLASQKRLENKYKAAEQASDDWYRRAQLALQKGDEDLAREALKRRKS
A. thaliana       108 NSDLTKMRQATAQVLASQKQLQNKYKAAQQSSDDWYKRAQLALAKGDEDLAREALKRRKS
P. trichocarpa    120 NDDLTKMRQATAQVLASQKRLENKYNATQQASEEWYRKAQLALQKGEEDLAREALKRRKT
G. max            100 NDDLTKMRQATAQVLASQKRLENKYKAAQQASEEWYRKAQLALQKGEEDLAREALKRRKS
S. lycopersicum   107 NNDLIKLRQATAQVLASQKQLENKYKAAQQASEDWYRRAQLALGKGDEDLAREALKRKKS
V. vinifera       102 NDDLTKMRQATAQVLASQKQLENRYKAAQQASEDWYKRAQLALGKGDEDLAREALKRRKS
consensus         121 * **.*.************ *.*.*.*..*.*..**..***** **.**********.*.

P. dactylifera    169 YADNASSLRAQLDQQKGVVDNLVSNTRLLESKIQEAKSKKDTLKARAQSAKTATKVSEML
O. sativa         154 YADNASSLKAQLDQQKGVVENLVSNTRVLESKIAEAKQKKDTLKARAQSAKTSTKVSEML
A. thaliana       168 FADNATALKTQLDQQKGVVDNLVSNTRLLESKIQEAKAKKDTLLARARTAKTATKVQEMI
P. trichocarpa    180 YADNANSFKAQLDQQRSVVENLISNTRLLESKIQEAKSKKDTLKARAQSAKTATKVNEML
G. max            160 YADNASSLKAQLDQQKTVVENLVSNTRLLESKIQEARSKKDTLKARAQSAKTATKVSEML
S. lycopersicum   167 YADNANALKTQLDQQKAVVDNLVSNTRLLESKIQEAKSKKDTLKARAQSAKTATKVSEML
V. vinifera       162 YADNASALKAQLDQQKTVVENLVSNTRLLESKIQEAKSKKDTLKARAQSAKTASKVSEML
consensus         181 .****. ...*****. **.**.****.*****.**..*****.***..***..**.**.

P. dactylifera    229 GNVNTSGALSAFEKMEEKVMAMESQAEALNQLTTDDLEGKFALLESSSVDDDLAKMKRDL
O. sativa         214 GNVNTSGALSAFEKMEEKVMAMESQAEALGQLATDDLEGKFALLETSSVDDDLAQMKKEI
A. thaliana       228 GTVNTSGALSAFEKMEEKVMAMESEADALTQIGTDELEGKFQMLETSSVDDDLADLKKEL
P. trichocarpa    240 GNVNTSNALSAFEKMEEKVLAMESEAEALGQLTTNELDGKFALLEGSSVDDDLENLKKEL
G. max            220 GNVNTSSALSAFEKMEEKVLTMESQAEALGQLTSDDLEGKFALLEGSSVDDDLANLKKEL
S. lycopersicum   227 GNVNTSSALSAFERMEEKVLTMESQADALNQLTSDELEGKFALLETSSVDDDLASLKKEL
V. vinifera       222 GNVNTSSALSAFEKMEEKVLAMESQAEALNQLTTDDLEGKFALLESSSVDDDLASLKKEL
consensus         241 *.**** ******.*****..***.*.** *.....*.***..**.*******. .*...

P. dactylifera    289 SGSSLRGELPAGRTAASSSNAAFPFRDIEIENELNELRRKAKDY
O. sativa         274 SGSSSKGELPPGRTAVSNSGAARPFRDIEIENELNELRKKANEY
A. thaliana       288 SGSSKKGELPPGRSTVA-ASTRYPFKDSEIENELNELRRKANDF
P. trichocarpa    300 AGSSKKGELPPGRTVVT--NSNNPFRDPDIEMELNELRQKRNNF
G. max            280 SGASKKGDLPPGRSTTS-ANTGIPFRDAEIELELDQLRQRAKEF
S. lycopersicum   287 SGSAKKGDLPPGRTPVTRSSSPLQFQDSEIEKELNELRRRANDF
V. vinifera       282 SGSSKKGELPPGRTAVAS-NTAFPFRDSEIEKELNELRRKASEF
consensus         301 .*....*.**.**. ..   .  .*.* .** **..**... ..
```

Supplementary Figure 3. Amino acid sequence alignment of plant VIPP1 orthologs.

Sequence alignment of full-length VIPP1 putative orthologs from species chosen as representative of the major angiosperm clades, as classified in Myburg *et al.* (2014). The species shown are: *Phoenix dactylifera* (*P. dactylifera*) XP_008788190, *Oryza sativa* (*O. sativa*) XP_015615370, *Arabidopsis*

thaliana (*A. thaliana*) NP_564846, *Populus trichocarpa* (*P. trichocarpa*) XP_006381403, *Glycine max* (*G. max*) NP_001242008, *Solanum lycopersicum (S. lycopersicum)* XP_004250100, and *Vitis vinifera* (*V. vinifera*) XP_002276816, which represent Arecales, Poales, Malvids, Malpighiales, Fabids, Asterids, and Vitales. Black or grey letters indicate identical and similar residues, respectively, which in the consensus line are highlighted with asterisks and periods. Other details are as described in the legend of Supplementary Figure 1.

A

SlVIPP1

1	MAVRTPITGITMSSAPSISSCNRVVVVKVMPFRASFFGQGVGAVKLAGLQ	50
51	LTHSYRSRCNSHGGGALGARMNLFDRFARVVKSYANALISTFEDPEKILE	100
101	QTVLEMNNDLIKLRQATAQVLASQKQLENKYKAAQQASEDWYRRAQLALG	150
151	KGDEDLAREALKRKKSYADNANALKTQLDQQKAVVDNLVSNTRLLESKIQ	200
201	EAKSKKDTLKARAQSAKTATKVSEMLGNVNTSSALSAFERMEEKVLTMES	250
251	QADALNQLTSDELEGKFALLETSSVDDDLASLKKELSGSAKKGDLPPGRT	300
301	PVTRSSSPLQFQDSEIEKELNELRRRANDF	350

VIPP1

1	MALKASPVTGLFPPLRPTASSSPSTSSNRPCSLRILPLRTSFFGNSSGAL	50
51	RVNVLRLACDNRLRCNGHGATMNLFERFSRVVKSYANALISSFEDPEKIL	100
101	EQTVIEMNSDLTKMRQATAQVLASQKQLQNKYKAAQQSSDDWYKRAQLAL	150
151	AKGDEDLAREALKRRKSFADNATALKTQLDQQKGVVDNLVSNTRLLESKI	200
201	QEAKAKKDTLLARARTAKTATKVQEMIGTVNTSGALSAFEKMEEKVMAME	250
251	SEADALTQIGTDELEGKFQMLETSVDDDLADLKKELSGSSKKGELPPGR	300
301	STVAASTRYPFKDSEIENELNELRRKANDF	350

B

SlVIPP1

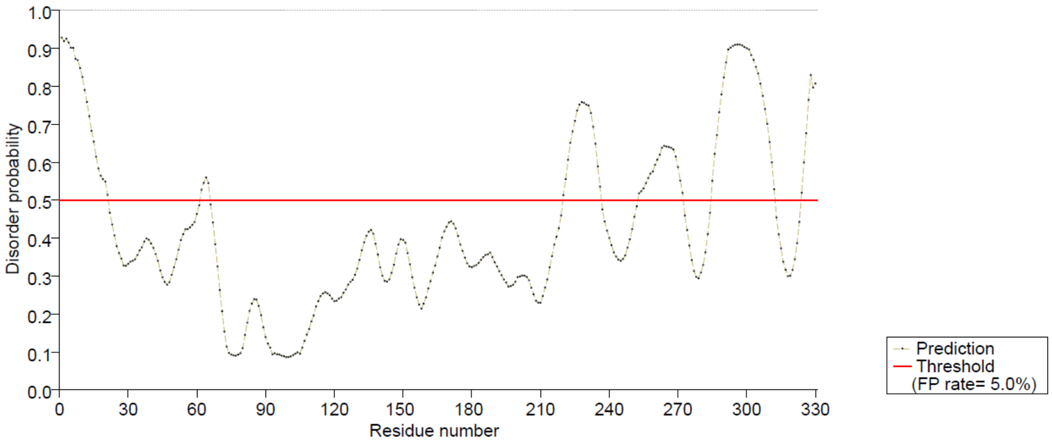

VIPP1

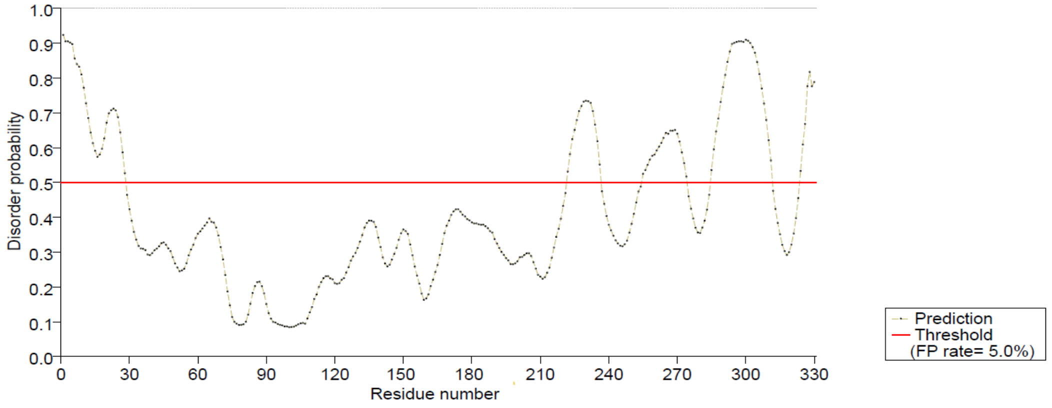

Supplementary Figure 4. Secondary structure prediction of tomato and Arabidopsis VIPP1 proteins. **(A)** PrDOS prediction of disordered regions in tomato and Arabidopsis VIPP1 proteins. Red amino acid sequences indicate predicted disordered regions. **(B)** PrDOS plot of disorder probability for each residue along the sequence of VIPP1. Residues above the red threshold line were predicted to belong to a disordered region.

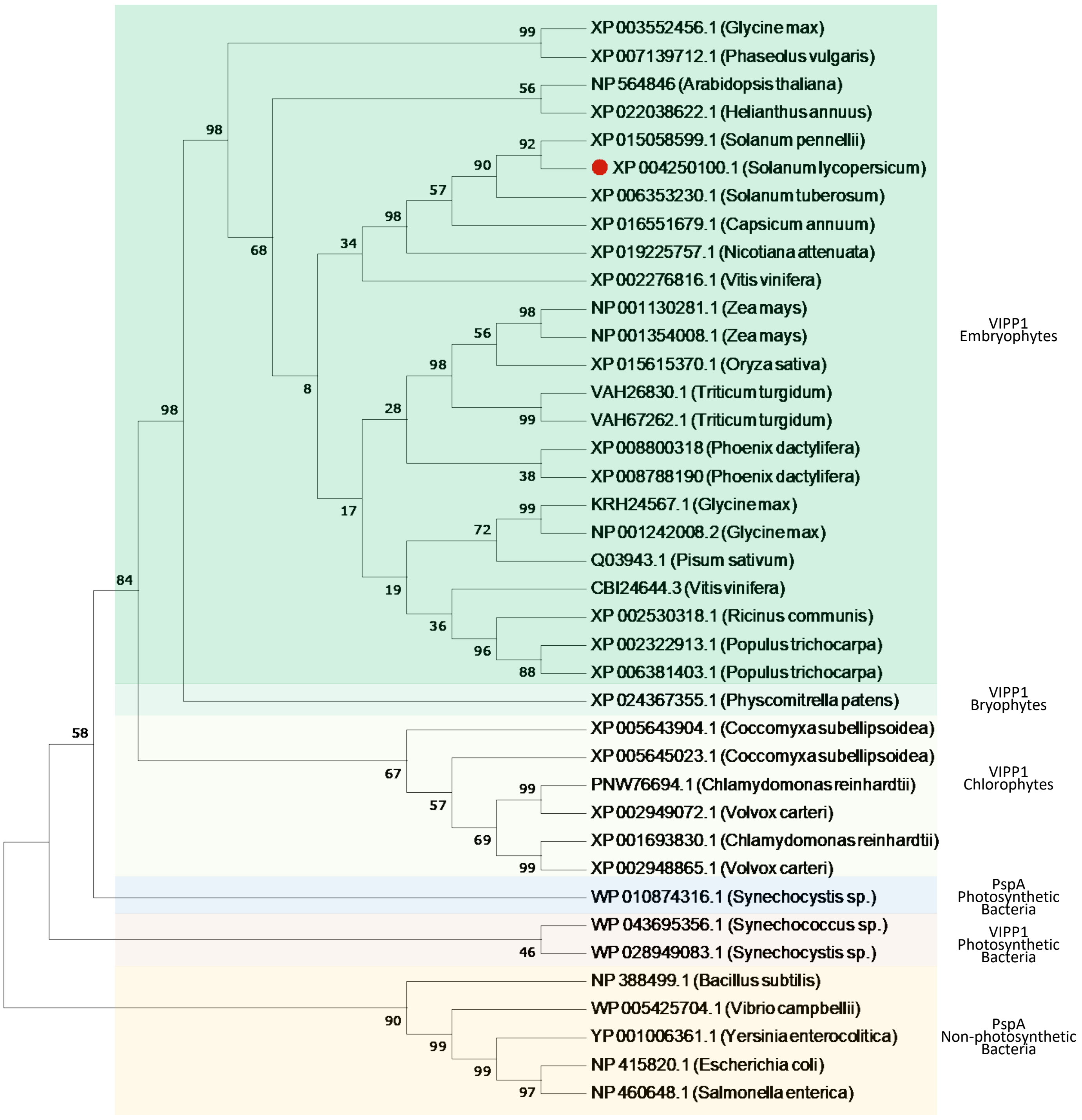

Supplementary Figure 5. Phylogenetic tree of VIPP1/PspA homologs from bacteria and green plants (Chlorophytes, Bryophytes and Embryophytes). The phylogenetic tree was constructed as described in Materials and methods. A red circle highlights the SLVIPP1 protein. GenBank accession numbers of the proteins are shown. Bootstrap values are indicated at tree nodes as percentages of 1000 replicates.
